# Thermal Balance in Male Water Buffaloes Transported by Long and Short Journeys

**DOI:** 10.3390/ani13203274

**Published:** 2023-10-20

**Authors:** Daniela Rodríguez-González, Isabel Guerrero Legarreta, Alfonso Chay-Canul, Ismael Hernández-Avalos, Fabio Napolitano, Ricardo García-Herrera, Alfredo M. F. Pereira, Adriana Domínguez-Oliva, Alejandro Casas-Alvarado, Brenda Reyes-Sotelo, Daniel Mota-Rojas

**Affiliations:** 1 Master in Science Program [Maestría en Ciencias Agropecuarias], Universidad Autónoma Metropolitana (UAM), Xochimilco Campus, Mexico City 04960, Mexico; 2 Department of Biotechnology: Food Science, Universidad Autónoma Metropolitana, Iztapalapa Campus (UAM-I), Mexico City 09340, Mexico; 3 División Académica de Ciencias Agropecuarias, Universidad Juárez Autónoma de Tabasco, Villahermosa 86040, Mexico; 4 Department of Biological Science, FESC, Universidad Nacional Autónoma de México (UNAM), Cuautitlán 04510, Mexico; 5 Scuola di Scienze Agrarie, Forestali, Alimentari ed Ambientali, Università degli Studi della Basilicata, 85100 Potenza, Italy; 6 Mediterranean Institute for Agriculture, Environment and Development (MED), Institute for Advanced Studies and Research, Universidade de Évora, 7006-554 Évora, Portugal; 7 Neurophysiology, Behavior, and Animal Welfare Assessment, Department of Animal Production and Agriculture (DPAA), Universidad Autónoma Metropolitana (UAM) Xochimilco Campus, Mexico City 04960, Mexico

**Keywords:** water buffalo, infrared thermography, journey time, thermostability, stress-induced hyperthermia

## Abstract

**Simple Summary:**

Livestock transport is a stressor with potential physiological, psychological, and financial consequences that can alter animal welfare, particularly in water buffalo, a species that has certain anatomical characteristics that make them susceptible to heat stress. Stress-induced hyperthermia is frequently observed in mobilized livestock, making it a parameter that could help to evaluate this event. Infrared thermography (IRT) is a tool that non-invasively assesses the thermal state of animals. Therefore, this study aimed to evaluate the surface temperature of water buffaloes monitored from the pasture to post-transport on short (SJs) and long journeys (LJs). When considering both groups, the highest temperatures were observed in the frontal-parietal region, while the lowest temperatures were registered in the nostrils. Moreover, a strong correlation was observed in all thermal windows. It is concluded that IRT can be used to accurately assess the thermal changes in buffaloes during transport.

**Abstract:**

Transport is a stressor that can cause physiological and metabolic imbalances in livestock, resulting in stress-induced hyperthermia. In water buffaloes, studies regarding the thermal state of animals during mobilization are scarce. Therefore, this study aimed to compare the thermal response of 1516 water buffaloes using infrared thermography (IRT) during 15 short trips (783 animals, 60,291 records, average duration = 50.33 min ± 5.48 min) and 14 long trips (733 animals, 56,441 records, average duration = 13.31 h ± 47.32 min). The surface temperature was assessed in 11 regions (periocular, lacrimal caruncle, nasal, lower eyelid, auricular, frontal-parietal, pelvic limb, torso, abdominal, lumbar, and thoracic) during seven phases from pasture to post-transport. It was found that the surface temperature of the periocular, lacrimal caruncle, nasal, auricular, frontal-parietal, pelvic limb, torso, abdominal, lumbar, and thoracic regions was significantly higher during SJs (+3 °C) when compared to LJs (*p* < 0.0001). In particular, the frontal-parietal region had a significant increase of 10 °C during the post-transport phase (*p* < 0.0001) in both groups, recording the highest temperatures during this phase. Likewise, a strong positive significant correlation between the different regions was found (r = 0.90, *p* < 0.0001). It is worth mentioning that the herding, loading, pre-, and post-transport phases were the ones where the greatest thermal response was recorded, possibly due to the influence of human interaction. Finally, a strong positive correlation (r above 0.9, *p* > 0.001) between the periocular, lacrimal caruncle, pinna, and pelvic limb was found. According to the results, SJ could be considered a stressful event that hinders thermal generation, contrarily to LJ.

## 1. Introduction

Animal transport is an almost mandatory event for livestock. It is recognized as a stressor due to several elements such as the type of vehicle, load density, type of road, vibrations, and the presence of unknown animals [1]. The frequency of injuries during transport is another issue that is more prevalent in water buffalo than in *Bos cattle* [2]. This might lead to the activation of the hypothalamic-pituitary-adrenal (HPA) axis [3,4,5,6]. The activation of this axis causes physiological alterations such as tachycardia, changes in the respiratory pattern, and metabolic modifications that can impact animal health and meat quality, making it necessary to evaluate the stress level that buffaloes might perceive during transport [3,4,5,7,8,9,10]. For example, increases in cortisol, lactate, serum protein, and hyperglycemia have been reported in cattle mobilized in larger distances of up to 16 h [11,12], together with behavioral alterations such as the increase in defecation, falls, and aggression [13,14]. As mentioned, the distance or duration of the journey is a critical factor that can hinder the health and mental state of animals when transported, causing weight loss and physical exhaustion [12], particularly when the type of vehicle and load density are not adequate for the species [15].

Currently, stress-related biomarkers are used to assess livestock welfare during transport such as blood or salivary cortisol levels. Nonetheless, it might not be easy to implement routine evaluations using these markers. As an alternative, infrared thermography (IRT) has been suggested to evaluate the surface thermal response of animals associated with stress. According to some studies, when an animal perceives a stressor, the activation of the HPA axis promotes catabolic metabolism and, consequently, an increase in body temperature [16,17]. The increase in core temperature can be reflected as a greater amount of radiated heat, an element that is detected through IRT. In water buffaloes, it has been reported that regions such as the ocular, pelvic limb, and frontal-parietal show increases between 3 and 5 °C after transport. This could be due to the limited thermoregulatory efficiency of water buffaloes due to the scarce presence of sweat glands, which makes them susceptible to heat stress or stress-induced hyperthermia [18].

Currently, there are no comparative studies on water buffalo assessing the effect of short and long journeys on the thermal state of buffaloes. Therefore, this study aimed to compare short (SJs) and long journeys (LJs) on the thermal response of the central and peripheral body regions of male water buffaloes, using IRT. It was hypothesized that LJs would record the highest temperature in central and peripheral regions, contrary to SJs.

## 2. Materials and Methods

### 2.1. Study Location

Short journeys (SJs) of water buffaloes were performed in south-southeast Mexico from June 2021 to August 2022. The average ambient temperature of the production unit where the animals were shipped was 31 ± 2 °C, with a relative humidity of 86%, 20 m.a.s.l., and annual rainfall of 1500–2000 mm (tropical humid climate). For long journeys (LJs), the buffaloes were monitored in the post-transport phase in the production unit of destination, having an average ambient temperature of 13.18 ± 1.42 °C, relative humidity of 63%, an elevation of 2807 m.a.s.l., and annual rainfall of 1679 mm (warm and temperate climate) [19].

### 2.2. Animals

SJs and LJs were classified according to their duration, where SJs had a mean duration of 50.33 ± 5.48 min and LJs had an average duration of 13.31 h ± 47.32 min. A total of 783 and 733 male buffaloes Buffalypso were included in SJs and LJs, respectively. For SJs, the animals were mobilized in 15 journeys, distributing the animals according to trucks’ the capacity of the first floor as follows: 41, 56, 46, 50, 48, 51, 57, 58, 53, 49, 52, 54, 56, 40, and 47. For LJs, the distribution during the 14 journeys was 53, 52, 55, 53, 54, 53, 51, 52, 53, 52, 53, 51, 51, and 50. For both groups, the inclusion criterion was that the selected animals had to be Buffalypso males with an approximate weight of 245 ± 19.36 kg, close to being mobilized to fattening and fattening units. On the contrary, clinically ill animals were considered an exclusion criterion.

All animals were from the same farm mentioned in subtopic 2.1. Regarding the distance traveled according to the groups, it was 27.5 km for SJs at an average speed of 55 km/h, while it was 732 km for LJs, with the same average speed. For both groups, water buffaloes were gently handled from the paddock without using physical tools or shouting that could cause injuries or pain.

### 2.3. Road Characteristics and Vehicle Type

The topography of the traveled roads for both SJs and LJs was classified as unpaved or paved according to the specifications described by Mexico’s Internal Ministry [20]. For unpaved roads, the classification was E and presented a maximum slope of 13%. During this route, a maximum speed of 25 km/h was reached, traveling 10 km of dirt roads for both SJs and LJs at the exit of the original production unit 6.3 km for SJs, and 13.2 km for LJs at the entrance of the reception production unit. On the other hand, for the paved road, a distance of 11.2 km was traveled in SJs and 718.8 km for LJs. This type of road had a maximum transversal slope of 7%; due to the characteristics of this road, a maximum speed of 55 km/h was reached. Vertical curves of 5m/% and a road of 8 m width was present in SJs and LJs.

Regarding the characteristics of the vehicle used, the truck had the following dimensions: 15.24 m long × 2.59 m wide × 4.6 m high. The truck had two floors of sliding side doors and a main door in the rear with a guillotine system. The walls were made of galvanized steel and aluminum, with ventilation openings, a non-slip floor, and reinforced plastic roof, and fiberglass.

### 2.4. Experimental Phases

The experimental protocol was divided in seven phases, described in Table 1.

### 2.5. Monitoring by Infrared Thermography

**Figure 1:**
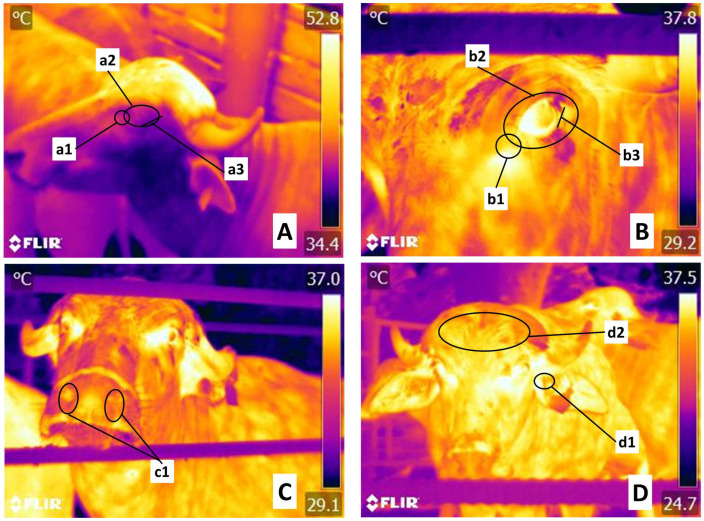
Thermal windows assessed in the head. (**A,B**). In the orbital region (*Regio orbitalis*), the lacrimal caruncle was delimited with a circle (**a1**,**b1**), from the medial commissure of the eyelids up to half a centimeter toward the cranium. The periocular thermal window (circles (**a2**,**b2**)) extends above the upper and lower eyelid. The lower eyelid (**a3**,**b3**) was defined by a line of approximately 3 cm, from which the conjunctival irrigation can be assessed. In the skull region (*Regiones cranii*) (**C**,**D**), nostril, auricular, and frontal-parietal windows were delimited. The nostrils (**c1**) were marked by ovals. For the auricular evaluation (*Regio auricularis*), a circle was used to mark the auditory canal (**d1**). Lastly, the frontal-parietal region (*Regio frontalis*) was delimited by a circle (**d2**), a zone irrigated by cornual and supraorbital arteries.

An infrared camera FLIR^®^ Thermal TM E60 (FLIR Systems, Wilsonville, OR, USA) was used. The camera had an IR resolution of 320 × 240 pixels, thermal sensitivity of <0.045 °C, and precision ± 2 °C or 2%. The thermal images were taken at an approximate distance of 1–1.5 m from the animals. After thermal imaging, the images were saved as JPG files to obtain the maximum, minimum, and average values using FLIR software (Version 6.4. FLIR Systems, USA). Six regions were evaluated: facial regions (*Regiones faciei*): the orbital region (*Regio orbitalis*) delimiting the lacrimal caruncle (1), periocular area (2), lower eyelid (*Regio palpebralis inferior*) (3), nasal region (*Regio nasalis*) (4). The skull regions (*Regiones cranii*) were the auricular (*Regio auricularis*) (5) and frontal-parietal region (*Regio frontalis-parietalis*) (6) (Figure 1).

**Figure 2:**
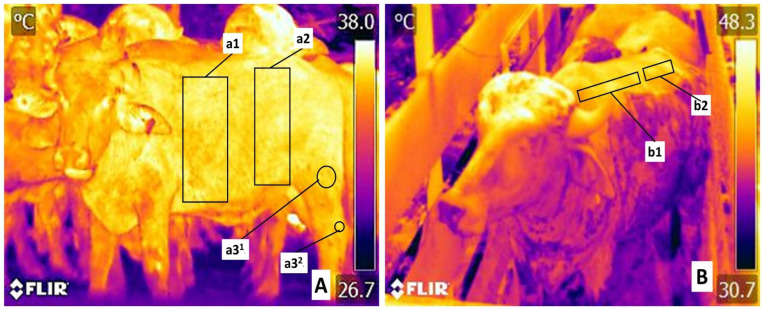
Evaluated thermal windows in the trunk region (*Truncus regionis*). (**A**) The coastal region (*Regio costalis*) was marked by a rectangle (**a1**) in the costal arch from 1° to 12° costal space. The cranial abdominal region (*Regio abdominis cranialis)* was delimited by a rectangle (**a2**) where the oblique abdominal and rectus abdominis muscles are present. The thermal window for the pelvic limbs (*Regiones membri pelvini*) was defined by two circles at the *Regio femoris* (**a3^1^**) and at the *Regio tarsi* (**a3^2^**), including the femoral muscle and the tarsus region. (**B**) Vertebral column (*Columna vertebralis*) thermal windows. The thoracic vertebral region (*Regio vertebralis thoracis*) is marked with a rectangle (**b1**), covering the thoracic vertebrae (1a to 12a) and the width of the transverse processes. The lumbar vertebral region (*Regio vertebralis lumbalis*) (**b2**) was delimited by a rectangle above the transverse processes of lumbar vertebrae L1–L7.

The trunk region (*Truncus regionis*), the thoracic (7) and abdominal (8) regions, and regions of the pelvis limb (*Regiones membri pelvini*) (9) were delimited with two anatomical regions indicating the femoral region (*Region femoris*) and the tarsus region (*Regio tarsi*) (Figure 2A). Figure 2B shows the last 2 thermal windows considered in this study: the thoracic vertebral region (*Regio vertebralis thoracis*) (10) and lumbar region (*Regio lumbalis*) (11).

Table 2 summarizes the experimental phases, its characteristics, hours, and ambient elements such as temperature and relative humidity.

### 2.6. Statistical Analysis

Statistical analyses were performed with GraphPad Prism (ver. 10.0.2; San Diego, CA, USA). Descriptive statistics were obtained for each thermal window (periocular, lacrimal caruncle, lower eyelid, auditory canal, nostrils, frontal-parietal area, and the thoracic, abdominal, appendicular, lumbar, and dorsal areas) and event (P1–P7). Values are expressed as mean ± standard error (SE). The Shapiro–Wilk test was used for normality analysis.

The independent variables were the events and thermal windows, while the surface temperature was considered as the dependent variable. An analysis of variance (ANOVA) in a linear mixed model was used as follows:

Y_ijak_ = µ+ τa + τi + τj + τk + τaτiτjτk + βk +e_aijk_

where:

Y = response variable (surface temperature)
τa = effect of the transport
τi = effect of the thermal window
τj = effect of the event
τk = effect of transport duration
τiτjτk = interaction effect
β = random effect (animal)
µ = population mean
e = error

To calculate the sample size, the G *Power program (ver. 3.1) was used considering an error probability of 0.05, a confidence level of 95%, a power (1-error probability) of 0.95 and a correction between repeated measures of 0.5 (total sample size = 700 animals per group) for two experimental groups with fixed effects, main effects and interactions of eleven thermal windows and seven event measurements.

A post-hoc Tukey test analyzed the differences between means. The level of significance was set at *p* < 0.05. Correlation analysis was conducted using the Pearson’s correlation coefficient.

### 2.7. Ethics Statement

The Scientific Commission of the Master’s in Science (CAMCA.11.21) “Maestría en Ciencias Agropecuarias” of the Faculty of Veterinary Medicine and Animal Husbandry, Universidad Autónoma Metropolitana, Mexico City, Mexico approved the methodology of the present research.

Animal ethical handling was performed following the Official Mexican Standards NOM-051-ZOO [22]. The animals included in the present study were not touched or stressed, since infrared thermography is a non-invasive technique.

## 3. Results

**Figure 3:**
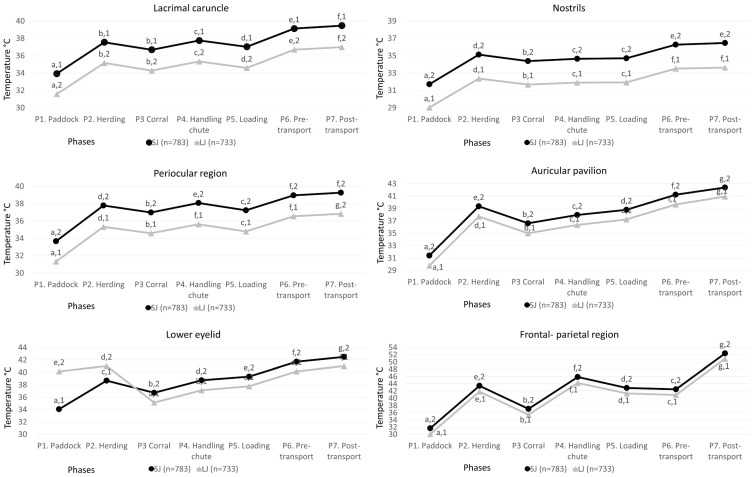
Mean ± standard error (SE) temperatures for the thermal windows of the head region (*Regiones capitis*) of SJ and LJ water buffaloes in the seven phases. Different literals ^a, b, c, d, e, f, g^ indicate statistically significant differences among the temperatures of the animals according to the phase (*p* value < 0.001). Different numbers ^1,2^ indicate statistically significant differences between short and long journeys of the thermal windows (*p* value < 0.001).

During the seven phases, 116,732 surface temperature readings were obtained from 11 thermal windows of 783 water buffaloes transported on SJs (50.33 ± 5.48 min) and 733 water buffaloes transported on JLs (13:31 h ± 47.32 min). Figure 3 shows the mean ± SE temperatures of the thermal windows in the head region (*Regiones capitis*).

The results obtained for the lacrimal caruncle in the SJ group show that the lowest temperature was recorded during P1, being 5.53 °C lower than P7, the phase with the highest temperature (*p* < 0.001). P2, P3, P4, and P5 did not have significant differences, with a maximum variation of 1.06 °C between P3 and P4 (*p* < 0.001). When comparing SJs and LJs, regardless of the phase, the highest temperatures were observed in SJs (*p* < 0.001), reporting the highest differences during P7 of up to 2.49 °C (*p* < 0.001).

For the periocular region, greater differences were observed in SJs. The lowest temperature was registered during P1 with a maximum variation of 5.59 °C in comparison to P7 (*p* < 0.001. This last phase was statistically similar to P6 with a minimum difference of 0.3 °C (*p* < 0.001). Regarding the LJ group, a maximum variation was observed between P1 and P7 (of up to 5.5 °C, *p* < 0.001), followed by the phases where human–animal interaction was present (P6, P4, P2, and P5).

Regarding the lower eyelid, SJs had the lowest temperature during P1, differing by 8.45 °C when compared with P7, followed by P6, P5, P4, and P2 (7.67, 5.25, 4.69, and 4.63 °C, respectively, *p* < 0.001). Regarding the LJ group, the lowest temperature was observed in P3 with a difference of 5.88° when compared with P7 and P2 (*p* < 0.001), followed by P1 and P6, a difference of 5.02; both were without numerical and statistical differences (*p* > 0.001). When comparing both groups, the greatest variation was observed in LJ buffaloes during P1, differing by 6.11 °C from SJ (*p* < 0.001).

For the nostril window in SJs, the greatest differences were observed during P1 and P7, with a statistically significant difference of 4.76 °C (*p* < 0.001). In contrast, similar values were maintained during P4 and P5 with minimum differences of 0.06 °C (*p* > 0.001). The highest temperatures were recorded in the phases where human interaction was present. For the LJ group, the greatest temperature variation was observed between P1 and P7 (4.61 °C, *p* < 0.001). In contrast to SJs, no significant differences were observed in the phases that had human interaction; this increase was only observed in phases 2, 6, and 7, (+3.33, +2.65, and +4.61 °C, respectively, *p* < 0.001). Regarding the differences between groups, the greatest numerical increase was observed in P7 in SJs, being 3.1 °C higher than LJ.

For the region of the skull (*Cranii regions*), specifically in the auricular thermal window, SJs had a marked difference and variation between phases. P1 presented the lowest temperature when compared with P7, recording a difference of 10.97 °C (*p* < 0.001), followed by P6, P2, and P5 with differences of 9.81, 7.95, and 7.38 °C, respectively (*p* < 0.001). As observed in the facial thermal windows (except the lower eyelid), a temperature drop in the LJ group was observed (*p* < 0.001). For the LJ group, there were differences in temperature of up to 13.16 °C when comparing P1 and P7 (*p* < 0.001). Regarding the differences found between groups, during P7, SJs differed by 1.45 °C from LJ animals.

Regarding the frontal-parietal region of the SJ group, marked differences were observed in all phases. P1 and P7 had differences of up to 20.7 °C, followed by phases with greater human handling (P4, P2, P5, and P6) with a temperature increase of 14.19, 11.78, 11.15, and 10.79 °C, respectively (*p* < 0.001). Regarding the LJ group, as in SJs, the lowest values were observed in P1; however, the variation was greater when compared with P7, with a difference of 20.86 °C (*p* < 0.001).

**Figure 4:**
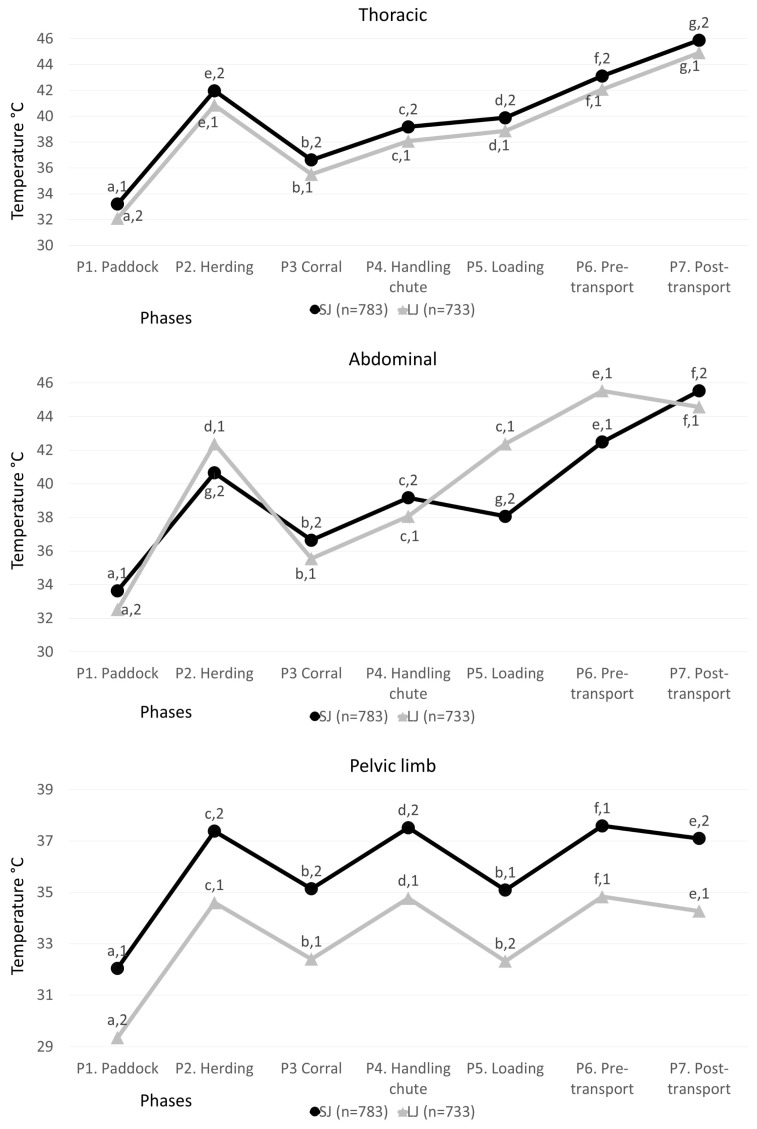
Mean ± standard error (SE) of the temperatures in the thermal windows of the lateral region of the trunk of SJ (short journey) and LJ (long journey) water buffaloes in the seven phases. Different literals ^a, b, c, d, e, f, g^ indicate statistically significant differences among the temperatures of the animals according to the phase (*p* value < 0.001). Different numbers ^1,2^ indicate statistically significant differences between short and long journeys of the thermal windows (*p* value < 0.001).

Figure 4 shows the temperatures regarding the results of the lateral region of the trunk divided into the thoracic and abdominal windows and the region of the extremities (pelvic limb).

Regarding the thoracic thermal window of the SJ group, significant differences were obtained in all phases of transport (*p* < 0.001), with the highest values in P7, P6, and P2, with differences of 12.67, 9.9, and 8.76 °C compared to P1 (*p* < 0.001) (lower values). Likewise, temperatures differed between P3 P2, and P4, where the animals had greater handling and movement by the operators (5.35 and 2.56 °C, respectively, *p* < 0.001). For the LJ group, the greatest variation between phases was also observed between P1 and P7, with an increase of 12.81 °C (*p* < 0.001) and an increase of 2.83 °C between pre- and post-transport (*p* < 0.001). Regarding the differences between groups (SJ and LJ), higher temperatures were observed in SJ buffaloes, with minimum differences of 0.97 °C (*p* < 0.001) in P7 and a maximum of 1.11 °C (*p* < 0.001) in P1 and P4.

For the abdominal thermal window, it was observed that SJ animals had the highest temperature during P7 and P6 with an increase of 11.89 and 8.85 °C, respectively, compared to P1 (*p* < 0.001). In phases where human–animal interaction was present, an increase of 2.52 °C was reported. In the LJ group, a different trend was observed from the rest of the thermal windows because the highest temperature was reported in P6 (13.01 °C higher than P1, *p* < 0.001). Regarding the comparison between SJ and LJ, differences were observed. P5 and P6 of LJ animals were higher than in SJ with differences of 1.01 °C (*p* < 0.001) and 2.84 °C (*p* < 0.001), respectively.

Regarding the pelvic limb of the SJ group, a similar trend was observed with the highest temperature recorded in P6, with an increase of 5.55 °C vs. P1 (*p* < 0.001), followed by P4, P2, and P7 increases of 5.48, 5.34, and 5.06 °C (*p* < 0.001). When comparing SJ and LJ, higher temperatures were observed in SJ regardless of the phase (*p* < 0.001), and higher variations were reported than in the lateral region of the trunk, with an average difference of 1.19 °C vs. 2.76 °C in the region of the extremities.

**Figure 5:**
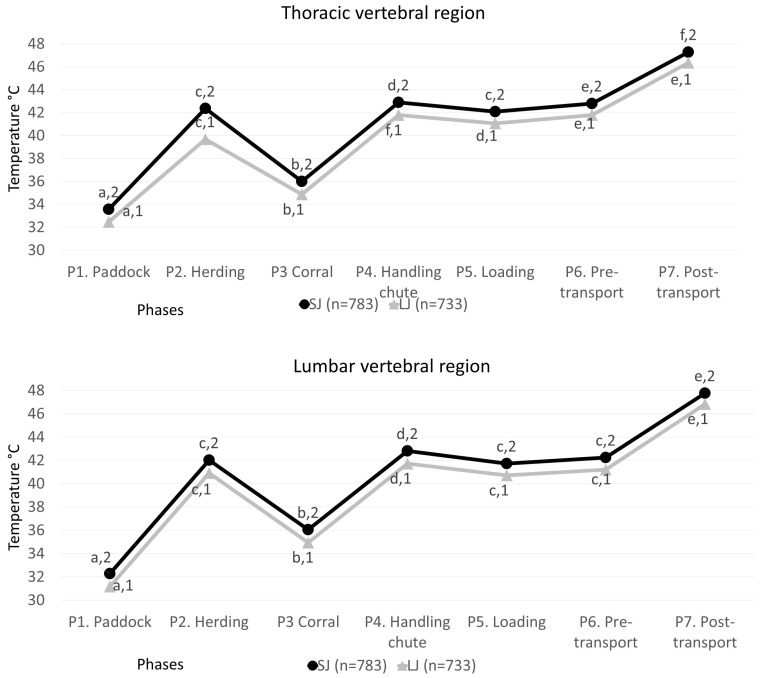
Mean ± standard error (SE) temperatures for the thermal windows of the trunk region (*Truncus regionis*) left or right of SJ (short journey) and LJ (long journey) water buffaloes in the seven phases. Different literals ^a, b, c, d, e, f,^ indicates statistically significant differences among the temperatures of the animals according to the phase (*p* value < 0.001). Different numbers ^1,2^ indicate statistically significant differences between short and long journeys of the thermal windows (*p* value < 0.001).

The thermal results obtained from the thoracic and lumbar vertebral region windows are seen in Figure 5.

For the thermal window of the thoracic vertebral region in the SJ group, a marked difference was observed between P1 and P7, with an increase of 13.72 °C (*p* < 0.001). Likewise, in P4 and P6, the values were statistically different, but they presented low numerical differences with a thermal drop of 0.1 °C (*p* < 0.001). In the LJ group, there were differences between all phases, recording the lowest temperature during P1, being 7.23 °C lower than P2 (*p* < 0.001), while P3 had a reduction of 3.36 °C when compared with P2 (*p* < 0.001) and increases of 2.11, 1.38, and 2.09 for P4, P5, and P6, respectively (*p* < 0.001). Regarding the comparison between groups, the lowest temperatures were observed in the LJ group with minimum numerical variations of 0.95 °C in P7 and a maximum of 2.67 °C in P2 (*p* < 0.001).

The values for the lumbar vertebral region showed that, for the SJ group, the temperature readings of P1 were the lowest (*p* < 0.001) with a variation of 9.74 °C compared with P2 (*p* < 0.001). P7 increased by 15.49 °C compared to P1 (*p* < 0.001). Regarding the LJ group, a marked difference of 15.68 °C was also observed in P7 with respect to P1 (*p* < 0.001). This trend was similar to the SJ group, with a variation at discharge from P1 to P2 of 9.76 °C (*p* < 0.001) and statistical similarity in P2 with P5 and P6 (*p* < 0.001). When making the comparison between groups, significant increases were observed in SJ (*p* < 0.001) with numerical variations from 0.93 °C in P7 (*p* < 0.001) to 1.12 °C in P1 (*p* < 0.001).

Table 3 reports the degree of correlation presented between the 11 thermal windows, having a positive and statistically significant correlation in all cases (*p* < 0.001).

## 4. Discussion

In general, it was observed that the surface temperature in the periocular, lacrimal caruncle, nasal, lower eyelid, auricular region, frontal-parietal, pelvic limb, torso, abdominal, lumbar, and thoracic regions was significantly higher (3 °C) during the herding, loading, handing chute, pre- and post-transport phases in SJs compared to LJs (*p* < 0.001). These findings show that animals might have presented a stress-mediated response. Moreover, shorter trips possibly increase the thermal response compared to longer ones [23].

The possible explanation for this is that SJs might initiate an acute stress response that can lead to the activation of the HPA axis and the secretion of glucocorticoids, triggering lipolysis and gluconeogenesis, which can generate problems in the quality of the meat due to the increase in pH [24,25] due to depletion of muscle glycogen; in the living animal with the increase in body temperature [26], it activates adaptive and compensatory mechanisms for restoring homeothermy and homeostasis [27]. Moreover, catecholamines are also released, increasing the metabolic activity of the heart, resulting in stress-induced hyperthermia during the first 10–15 min after exposure to the stressor [28,29] in preparation for the possibility of rapid energy expenditure [30]. In this regard, the secretion and action of catecholamines and the stress induced by hyperthermia also have effects on muscle metabolism and membrane integrity, generating the appearance of undesirable characteristics in meat [31] and the decrease in live weight and the modification of physicochemical characteristics that affect both the quality and safety of the final cut [32]; the above is mainly associated with the depletion of muscle glycogen reserves and the accumulation of ante-mortem lactic acid, increasing post-mortem pH values [33], cooking loss, meat hardness [34], and ageing potential [35].

This coincides with what was reported by Burdick et al. [36], who found that the highest rectal temperature of Brahman bulls was recorded within the first 30 min after transport, while the lowest temperature occurred at 6 h and 40 min after transport. In this sense, SJs might not give enough time for the animals to habituate, culminating in an acute stress response.

For LJs, the buffaloes might be able to habituate to transport and its related stressors based on what has been indicated in previous studies [37,38]. This coincides with what was observed by Lei et al. [39], where they found that 15 to 17 h transport of 20 Arouquesa calves had an initial increase of 3 °C in periocular temperature and a subsequent decrease of 2 °C. The authors concluded that the animals habituated to the transport, although the increase in temperature represents an important metabolic cost, which can be difficult to maintain over a long period as represented by the LJ group. According to the findings of the present study and already published studies, the proposed hypothesis can be rejected.

Another element related to the thermal response observed in LJ animals would be the secretion of catecholamines, which leads to physiological effects such as tachycardia and vasoconstriction [40]. Therefore, a decrease in heat radiation may occur, associated with an increased sympathetic response as has been observed in cattle under conditions associated with acute pain [41,42,43]. Thus, it is possible to suggest that although the surface temperature was 5 °C lower in the animals from the LJ group, the thermal response may be associated with a chronic event compared to the animals in the SJ group. It should be noted that this consensus is difficult to affirm due to the lack of evidence because other factors such as the decrease in visceral activity due to prolonged fasting on long-distance trips and decreased body temperature could also influence the thermal response [44,45].

On the other hand, it was observed that the surface temperature in both SJs and LJs, the herding, handling chute, and pre-and post-transport phases significantly increased by at least 3 °C compared to the rest of the phases (*p* < 0.001). The explanation for these findings is that in these phases, interaction with humans is involved [46], which can often be negative due to the shouting, hitting, or the use of sticks and electric prods that generate pain and fear in the animal, increasing the acute stress response [47]. It is mentioned that mobilization and human–animal interaction without prior training are stressful for livestock, as they involve both physical and psychological aversive stimuli [3]. These results coincide with those reported by Rodríguez-González et al. [9], where they observed that the surface temperature in 624 water buffaloes mobilized in SJs (average duration = 2 h ± 20 min) had an average increase of 5 °C during the phases, where some type of handling was present. Then, it is possible to reaffirm that these interactions can cause acute fear and a physiological response associated with this handling, limiting the productivity, performance, and animal welfare [48].

From a neurobiological perspective, fear is processed by brain regions such as the somatosensory cortex, hippocampus, thalamus, and amygdala, which in coordination with the latter structure process the fear response (e.g., tachypnea, tachycardia, or hyperthermia) [49,50,51]. This response may explain the increase in surface temperature in the evaluated thermal windows due to the activation of HPA [52] and the subsequent secretion of endogenous glucocorticoids [50]. A possible additional explanation is neophobia, a trait present in water buffaloes, which possibly makes them susceptible to fear of unknown environments such as transport [53]. In this sense, it has been reported that dairy cattle may experience more fear in unfamiliar milking parlors and stocks [54].

The same response associated with fear may explain the differences found between the thermal windows evaluated in the present study. Perhaps the clearest example could be observed in the nasal window, where the thermal response serves to evaluate the physiological parameter of the respiratory frequency [54,55]. Considering that tachypnea is one of the physiological responses associated with stress, this can lead to an increase in radiated heat [56].

Regarding the differences found between groups in the facial region, greater imbalances were present in the SJ group between P1 and P7 in the lower eyelid window. This greater variation was also present in the rest of the thermal windows of the facial region for this group. The changes in the temperature of the facial region may be related to parasympathetic activity, the vasodilation response, which generates a lowers cardiac output and blood pressure and the consequent increase in temperature [39]. In particular, the wide distribution of capillaries and arteriovenous anastomoses in these thermal windows facilitates the exchange of body heat with the environment [57].

In another way, the lowest values were observed in the nasal window of the LJ group during P1 (29.03 ± 0.04 °C, *p* > 0.001). The above may be due to the nature of the anatomical region itself, which presents a high superficial vascularization from the maxillary vein and artery [58], in addition to the humidity given by the water vapor eliminated during respiration, which is affected by the stress generated during tachypnea [54]. Other authors also describe changes in temperature coming from the nasal region as a reflection of the activity of the autonomic nervous system, which can be used to detect an increase in the level of arousal (with a decrease in temperature) due to increased sympathetic activity and decreased blood flow in peripheral vessels [59,60].

Regarding the regions of the skull and their respective thermal windows, a greater variation was observed between groups. This region receives irrigation by the corneal artery, the superficial temporal artery (*A. temporalis superficialis*), and its branches (*A. transversa faciei, A. auricularis rostralis, A. palpebralis inferior lateralis, A. palpebralis superior lateralis*) [61]. In this sense, Badakhshan et al. [62] evaluated the temperature of Jersey cattle in different anatomical regions and its correlation coefficient to the rectal temperature, respiration rate, and heart rate. The authors observed that forehead values had a strong, positive, and significant correlation (r = 0.57, r = 0.88, and r = 0.70 respectively, *p* > 0.01). It has also been specified that, in bovid species raised in arid areas, the horn has thinner keratin sheaths than in temperate climates to facilitate heat loss [63,64]. Algra et al. [65] supported the role of horns for thermoregulation in dairy cattle, monitoring dehorned cows using IRT. These animals presented higher eye temperatures than horned cows.

Regarding the body regions specified in Figure 4, a similar trend was observed in the thoracic thermal window and the pelvic limb, with higher thermal variations in the pelvic limb. On the contrary, the thoracic thermal window presented smaller variations with minimums of 0.97° and maximums of 1.11 °C in phases 7 and 1–4, respectively (*p* < 0.001). With the exception of the abdominal thermal window, LJ group reported the highest temperatures without considering the phase or the window. The behavior of the thoracic and abdominal regions may be due to the presence of metabolically active organs. Furthermore, it has been established that the relationship between the environmental and surface temperature of animals is usually closer to that of the thorax, where the variation in blood is minimal [66], due to the irrigation of the caudal aorta artery (*A. aorta abdominalis*) towards abdominal organs and the cranial, median, and caudal quadrants [67].

Conversely, the lowest surface temperatures for all phases were observed in the pelvic limb and, in this same window, a greater variation was observed between groups (*p* > 0.001). This was also observed in a recent study conducted on 109 newborn water buffalo calves, who found the lowest temperature values in the pelvic limb compared to thermal windows of the facial region (lacrimal caruncle, periocular region, and lacrimal gland) [68]. The above could be explained due to the reduction in blood flow due to vasoconstriction and closure of arteriovenous anastomoses of extremities during stressful processes such as transport [69], prioritizing blood supply to the thoracic and abdominal area.

Finally, Table 3 shows the level of correlation between both central and peripheral windows being positive, strong, and significant (r above 0.9, *p* > 0.001). This means that, with an increase in body temperature, the surface temperature of the animals might also increase [9,16,70]. The above could be due to the effect of glucocorticoids that trigger the degradation of lipids and glycogen, affecting the temperature of both central and peripheral windows, with a greater increase in those thermal windows close to metabolically active organs [26,71].

The analysis carried out in this study represents novel information regarding the thermal behavior and the effect of the time factor during the transport of water buffaloes monitored by IRT. However, it is necessary to make visible the limitations of this article by not evaluating other physical variables such as rectal temperature, heart, and respiratory rate, as well as blood concentrations of cortisol, glucose, lactate, and pH values, or biomarkers related to dehydration processes (osmolarity, albumin, and hematocrit) and other biomarkers such as catecholamines, alpha amylase, IL, TNF alpha, and creatine kinase, among others, as well as the evaluation of facial expressions, behavior, and emotions, which opens a range of future research regarding the effect of transportation and the handling that this implies on physiological and thermal indicators in water buffalo.

Although the present study compared the thermal response before and after short and long journeys, a limitation of this study and a field of research for future studies is the consideration of the thermal response of buffaloes during the journey. Furthermore, changing climatic conditions between a short and a long journey, such as ambient temperature or relative humidity, need to be considered when interpreting infrared imaging. This is relevant because ambient temperature affects animals’ surface temperature [72]. Nonetheless, although environmental factors might have some influence on thermoregulation, the findings of the present study show that transport is a potential stressor that alters the thermal response of buffaloes [73,74]. Further research should adopt and consider these variables to comprehend the influence of said variables in the possible stress-mediated thermal response of water buffaloes.

## 5. Conclusions

In conclusion, short journeys increase the thermal response in water buffaloes compared to long journeys monitored by IRT, and this could be associated with acute stress, refuting the proposed hypothesis. This could be because, during LJ, water buffaloes can become accustomed to stressors. Likewise, it is concluded that the phases that involve human–animal interaction generate an increase in the surface temperature for all the thermal windows evaluated, reaffirming the importance of adequate management during the transportation process to avoid negative effects and surface temperatures of the mobilized buffaloes.

This study is of utmost importance as it is a pioneer in the characterization and analysis of the thermal behavior of water buffalo during long journeys.

## Figures and Tables

**Table 1:** Description of the characteristics of each evaluation phases in both SJs and LJs.

Phases	Description	Characteristics
P1	Paddock	Considered as basal monitoring. The animals were housed in paddocks whose feeding system is based on pastures, providing 50 g of mineral supplements per animal. In each paddock, the presence of native grasses (*Paspalum fasciculatum*) (dry matter = 14.7%, 56.7% neutral detergent fiber, 38.8% acid detergent fiber and 6.2% raw protein [21].
P2	Herding on horseback	Animals with and appropriate body weight were selected. Handlers moved the buffaloes with the help of horses and without the use of physical tools that could cause injuries. For both SJs and LJs, the average duration of this phase was between 25 and 35 min.
P3	Corral system	Once the animals entered the pen, they remained for 12 consecutive hours (including the night before weighing, handling, and transport). The pen surface area was 630 m2 with a dirt floor. It was delimited by metal structures with 1.6 m height and two doors, of which one connected to the handling chute (P4). The animal did not receive solid food during the last 8 h but had water ad libitum.
P4	Handling chute	The handling chute hallway dimensions were 1.6 m 1 m wide × 1.6 m height × 3.5 m long. It was connected to an individual weight scale and a loading ramp. The individual passing of animals through the ramp had an average duration of 50 min.
P5	Loading	Buffaloes were monitored for 30–50 min during their passage on a loading ramp with anti-slip grooving with a slope of 20°.
P6	Pre transport	Monitorization was performed after the animal was loaded into the truck, inside the vehicle.
P7	Post transport	Thermal monitoring was carried out when the animals were still inside the vehicle, in the shade, after arriving at the reception site.

**Table 2:** Specifications of each experimental phase (hours, ambient temperature, and relative humidity) for both SJ and LJ.

Phases	Description	Hours	Ambient Temperature (AT) and Relative Humidity (RH)
P1 Paddock	Prior to the buffaloes being herded by men on horseback, while they were at rest for one hour under shade	Between 17:00 and 18:00	AT-23–25 °C RH-81–90%
P2 Herding	Inside the corral on the day prior to transport	Between 18:00 and 18:30	AT-21–25 °C RH-81–88%
P3 Corral	Before entering the handling chute to be weighed and loaded	08:00	AT-20–23 °C RH-81–86%
P4 Chute	While the buffaloes were inside the handling chute	09:00	AT-22–25 °C RH-81–88%
P5 Loading	During loading, with the buffaloes walking up the ramp	10:00	AT-22–27 °C RH-81–88%
P6 Pre transport	After loading the truck, with the motor shut off	11:00	AT-22–28 °C RH-81–89%
P7 Post transport	Immediately after reaching the destination for both short and long trips, with the truck’s motor still turned on	SJ-11:50 ± 5.48	AT-23–30 °C RH-83–94%
		LJ-00:31 ± 47.32	AT-10.5–15.7 °C RH-84–46%

**Table 3:** Correlations between the thermal windows of SJ (short journey) and LJ (long journey) water buffaloes in the seven phases.

	Group	Lacrimal Caruncle	Periocular Region	Lower Eyelid	Auricular Pavilion	Nostrils	Frontal-Parietal Region	Thoracic Region	Abdominal Region	Limbs Region	Lumbar Region	Thoracic Vertebral Region
Lacrimal caruncle	SJ (*n =* 7383)	0.935 *										
	LJ (*n =* 733)	0.935 *										
Periocular region	SJ (*n =* 7383)	0.963 *	1 *									
	LJ (*n =* 733)	0.989 *	0.999 *									
Lower eyelid	SJ (*n =* 7383)	0.971 *	0.998 *	1 *								
	LJ (*n =* 733)	0.976 *	0.974 *	0.999 *								
Auricular pavilion	SJ (*n =* 7383)	0.941 *	0.997 *	0.974 *	1 *							
	LJ (*n =* 733)	0.811 *	0.839 *	0.801 *	0.999 *							
Nostrils	SJ (*n =* 7383)	0.962 *	0.999 *	0.992 *	0.999 *	1 *						
	LJ (*n =* 733)	0.989 *	0.989 *	0.999 *	0.984 *	0.999 *						
Frontal-parietal region	SJ (*n =* 7383)	0.957 *	0.999 *	0.991 *	0.981 *	0.999 *	1 *					
	LJ (*n =* 733)	0.976 *	0.977 *	0.990 *	0.964 *	0.971 *	0.999 *					
Thoracic region	SJ (*n =* 7383)	0.954 *	0.988 *	0.995 *	0.987 *	0.999 *	0.999 *	1 *				
	LJ (*n =* 733)	0.981 *	0.977 *	0.993 *	0.965 *	0.974 *	0.999 *	0.999 *				
Abdominal region	SJ (*n =* 7383)	0.952 *	0.989 *	0.987 *	0.963 *	0.986 *	0.999 *	1 *	1 *			
	LJ (*n =* 733)	0.967 *	0.953 *	0.989 *	0.948 *	0.986 *	0.988 *	0.999 *	0.999 *			
Limbs region	SJ (*n =* 7383)	0.952 *	0.999 *	0.988 *	0.999 *	0.999 *	0.987 *	0.988 *	0.975 *	1 *		
	LJ (*n =* 733)	0.996 *	0.999 *	0.999 *	0.999 *	0.976 *	0.999 *	0.999 *	0.982 *	0.999 *		
Lumbar region	SJ (*n =* 7383)	0.957 *	0.999 *	0.988 *	0.986 *	0.999 *	0.999 *	0.999 *	0.999 *	0.999 *	1 *	
	LJ (*n =* 733)	0.982 *	0.975 *	0.999 *	0.978 *	0.987 *	1 *	0.987 *	0.994 *	0.976 *	0.999 *	
Thoracic vertebral region	SJ (*n =* 7383)	0.945 *	0.982 *	0.999 *	0.975 *	0.983 *	0.999 *	1 *	0.999 *	0.985 *	1 *	1 *
	LJ (*n =* 733)	0.975 *	0.974 *	0.999 *	0.963 *	0.978 *	0.999 *	0.999 *	0.999 *	0.978 *	0.999 *	0.999 *

* *p* < 0.0001.

## Data Availability

Not applicable.
